# New Zealand’s new alcohol laws: protocol for a mixed-methods evaluation

**DOI:** 10.1186/s12889-015-2638-9

**Published:** 2016-01-13

**Authors:** Brett Maclennan, Kypros Kypri, Jennie Connor, Tuari Potiki, Robin Room

**Affiliations:** 1 Department of Preventive and Social Medicine, University of Otago, PO Box 56, Dunedin, 9054 New Zealand; 2 School of Medicine and Public Health, University of Newcastle, Newcastle, New South Wales Australia; 3 Office of Māori Development, University of Otago, Dunedin, New Zealand; 4 Centre for Alcohol Policy and Research, La Trobe University, Melbourne, Australia; 5 Centre for Social Research on Alcohol and Drugs, Stockholm University, Stockholm, Sweden

**Keywords:** Alcohol policy, Evaluation, Community participation, Alcohol availability, Alcohol-related harm, New Zealand, Local government

## Abstract

**Background:**

Alcohol consumption is a major cause of mortality and morbidity globally. In response to strong calls from the public for alcohol law reform, the New Zealand Government recently reduced the blood alcohol limit for driving and introduced the Sale and Supply of Alcohol Act which aim to (1) improve community input into local decision-making on alcohol; (2) reduce the availability of alcohol; and (3) reduce hazardous drinking and alcohol-related harm. In this project we seek to evaluate the new laws in terms of these objectives.

**Design and methods:**

A policy evaluation framework is proposed to investigate the implementation and outcomes of the reforms. We will use quantitative and qualitative methods, employing a pre-post design. Participants include members of the public, local government staff, iwi (Māori tribal groups that function collectively to support their members) and community group representatives. Data will be collected via postal surveys, interviews and analysis of local government documents. Liquor licensing, police and hospital injury data will also be used. Community input into local government decision-making will be operationalised as: the number of objections per license application and the number of local governments adopting a local alcohol policy (LAP). Outcome measures will be the ‘restrictiveness’ of LAPs compared to previous policies, the number (per 1000 residents) and density (per square kilometre) of alcohol outlets throughout NZ, and the number of weekend late-night (i.e., post 10 pm) trading hours. For consumption and harm, outcomes will be the prevalence of hazardous drinking, harm from own and others’ drinking, community amenity effects, rates of assault, and rates of alcohol-involved traffic crashes. Multiple regression will be used to model how the outcomes vary by local government area from before to after the law changes take effect. These measures will be complemented by qualitative analysis of LAP development and public participation in local decision-making on alcohol.

**Discussion:**

The project will evaluate how well the reforms meet their explicit public health objectives.

## Background

The purpose of the Evaluation of New Zealand’s Alcohol Laws (ENZAL) project is to evaluate the effectiveness of recent law changes in terms of the objects of the legislation, namely, to: 1) improve community input into local decision-making on alcohol availability, 2) reduce the availability of alcohol, and 3) reduce hazardous drinking and alcohol-related harm. Though the laws are national, their provisions mean that effects may vary by locality. ENZAL thus draws on multiple methods and strategies of analysis to evaluate effectiveness both at national and local levels. Results will inform future alcohol policy development and implementation, and will contribute to an international evidence base on the effects of alcohol policy.

### The burden of harm

Alcohol consumption is a prominent cause of death, disease and injury globally. It is the 8th leading risk factor for mortality and ranks 5th for morbidity, accounting for 2.7 million deaths and 4 % of disability-adjusted life years (DALYs) lost [1]. In New Zealand (NZ), among those aged less than 80 years, alcohol causes 5.4 % of deaths and 6.5 % of DALYs lost [2]. Two-thirds of these alcohol-related deaths are in men. Māori, the indigenous population of NZ, have an age-standardised alcohol-attributable death rate 2.5 times higher than non-Māori [2].

### Strategies to reduce alcohol consumption and harm

The most recent in a series of comprehensive literature reviews sponsored by the World Health Organisation [3] concluded that policy interventions restricting the availability and promotion of alcohol were the most effective approach to reducing alcohol consumption and related harm, in contrast to education campaigns and other methods of persuasion. Effective countermeasures include restrictions on the density and trading hours of alcohol outlets, a minimum legal drinking or purchase age of 20 or 21 years, and higher excise taxes. A blood alcohol limit for driving of 0.05 g/dL or less, which reduces the demand for alcohol in highly motorised countries, is also an effective strategy [4].

### Alcohol policy in New Zealand

Starting with the 1989 Sale of Liquor Act, successive NZ governments liberalised alcohol policy [5]. Deregulation left market forces to determine the number of licensed premises and permitted supermarkets to sell wine and beer. Consequently, the number of alcohol outlets increased from 6000 to 14,000 in a decade [5, 6]. This led to aggressive competition resulting in extensive marketing and heavy discounting [7]. Other law changes provided for broadcast advertising of alcohol, increased trading hours, alcohol sales on Sundays, and for the minimum purchasing age to be reduced from 20 to 18 years [7].

### Law commission review

The increase in alcohol availability and promotion since 1989 has been associated with an increase in harm [8–10]. Public concern over the growing harm motivated a comprehensive review of the country’s alcohol laws by the Law Commission [7]. Its final report drew from research evidence and 3000 public submissions to produce recommendations for substantial legislative reform [11, 12].

### The sale and supply of alcohol Act (2012)

The Government responded to the Law Commission’s review by passing the Sale and Supply of Alcohol Act (SSAA). In addition to minimising harm from alcohol [13], a major focus of the Act is on giving communities more say in how alcohol is sold locally. This is to be facilitated by local governments, i.e., territorial authorities (TAs), through the development of Local Alcohol Policies (LAPs), a newly developed mechanism to enable local input.

The Sale of Liquor Act (1989) intended to facilitate greater community control by devolving liquor licensing to TAs [5]. At that time, some TAs adopted policies specifying trading hours and restrictions on outlet locations [14]. However, unlike LAPs under the new SSAA (2012), these carried no statutory weight. Other legislative constraints rendered licensing authorities powerless to address outlet density and community concerns about cumulative impacts on amenity and health [7, 15, 16]. As a result, communities throughout NZ have become increasingly frustrated over the inability to have meaningful input to how alcohol is sold locally [11].

The SSAA permits LAPs to regulate both outlet density and hours of sale [4, 17–19]. The changes are intended to meet the Act’s object to “minimise the harm caused by the excessive consumption of alcohol” [13] and its wider purpose to “improve community input into local alcohol licensing decisions” [20]. However, the process for developing a LAP is complex and may be hindered by appeals and Court proceedings (e.g., [21]). This may lead to an increase in alcohol availability in some communities as TAs that do not adopt a LAP will be subject to the default trading hours (up to 4 am).

The Act also broadens the grounds on which the public can object to a licence application, and for authorities to refuse to grant a licence. These include arguments that the amenity of the locality would be reduced, or that inconsistencies with a LAP would arise from granting the licence.

Community mobilisation efforts could be an important factor in whether or not the reforms lead to reductions in alcohol availability, consumption and related harm. There is some evidence that community action can lead to a reduction in alcohol problems but these efforts are resource intensive and reductions in harm can be short-lived [4, 22].

Provisions concerning objections to and refusal of licences took effect on 18 June 2013. The majority, including those concerning LAPs, came into effect 6 months later. This meant that the earliest a TA could adopt a LAP, provided no appeals were lodged, was 18 January 2014. As of October 2015, three of 67 TAs have a LAP in force.

### Amendments to the land transport Act (1998)

Another law change supported by the Law Commission but outside the remit of their review was a reduction in the blood alcohol limit for drivers aged ≥20 years. In a process separate from the SSAA, in December 2013 the Government reduced the legal limit from 0.08 to 0.05 g/dL. The new limit came into effect on 1 December 2014. The legal limit for drivers under 20 was already zero.

## Aims

The aims of ENZAL are to evaluate the effectiveness of the new laws in: 1) improving community input into licensing decisions for Māori and non-Māori; 2) reducing the availability of alcohol in NZ communities; and 3) reducing hazardous drinking and alcohol-related harm among Māori and non-Māori.

## Design and methods

**Fig. 1:**
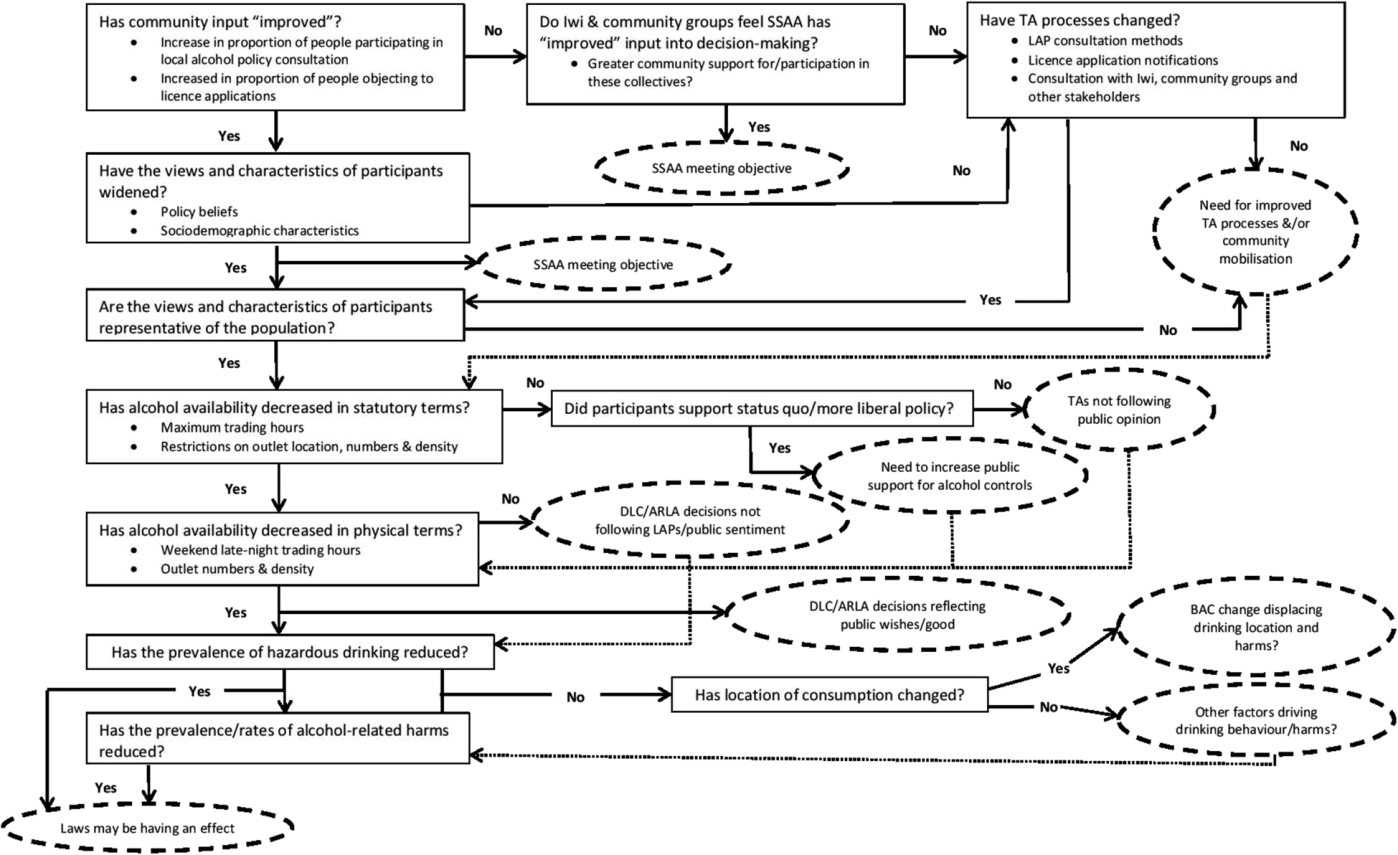
Evaluation framework

A policy evaluation framework, focussing on implementation of the new laws and the outcomes of those changes, will guide the research (Fig. 1) [23]. Community surveys, key informant interviews and administrative data will be used to evaluate community uptake of the new opportunities provided by the SSAA and the impact on alcohol availability, consumption and harm. The project consists of three parts (Table 1) corresponding to the study aims, and will take 4 years, allowing time for short-term effects to be assessed. Ethical approval for ENZAL was granted by the University of Otago Human Ethics Committee (D14/290).

**Table 1:** Evaluation elements

PART 1 Policy objective: Improve public input into licensing decisions		
Measure	Data	Analysis
• Change in proportion of residents participating in local decision-making	National surveys 2014 & 2017	Comparison of proportions (*χ* ^2^ tests)
• Change in objections per application for licence to sell alcohol	TA & ARLA data	Negative binomial regression
• Influences on and satisfaction with local decision-making	Interviews with iwi and community groups, telephone survey of TAs, document analysis	Qualitative
• Number of LAPs adopted/being developed by mid-2017	ARLA registry, telephone survey of TAs	Descriptive, logistic regression
PART 2 - Policy objective: Reduce alcohol availability		
Measure	Data	Analysis
Change in:		
• outlet numbers and density	Geographic information system (Ministry of Justice data)	Negative binomial/Poisson regression
• maximum trading hours permitted and total weekend late-night trading hours	Telephone survey of TAs, document analysis, Ministry of Justice data	Descriptive and Negative binomial/Poisson regression
• use of one-way door restrictions	Telephone survey of TAs, document analysis	Descriptive
PART 3 - Policy objective: Minimise alcohol-related harm		
Measure	Data	Analysis
Change in the prevalence/incidence of:		
• hazardous drinking & personal adverse effects	AUDIT & harm items in national surveys 2014 & 2017	Comparison of proportions (*χ* ^2^ tests)
• secondhand & community amenity effects	SHE & CAE items in national surveys 2014 & 2017	
• late-night assaults per month	Police data	Negative binomial/Poisson regression
• weekend hospitalisations for assault per month	National Minimum Dataset	
• alcohol-involved traffic crashes per month	Police data	

### Part 1: community input

Part 1 addresses the first aim of the study, to determine whether the SSAA improves community input into local decision-making on alcohol. The legislature was not specific about what “improving” community input means. We have operationalised it as:

- increases in the proportion, and widening of the characteristics and views, of people who participate in local alcohol decision-making;
- groups involved in local policy action on alcohol indicating that the new law has facilitated their input into decision-making;
- the development of LAPs with more inclusive consultation methods compared with those used to develop previous alcohol policies; and
- increases in the number of LAPs including among communities with lower average socioeconomic status (Table 1).

The complementary components of Part 1 will reveal whether more people are participating, how representative participants are of the general public, and whose input has influenced the development and adoption of LAPs and licensing decisions. Part 1 will allow us to assess whether the new law is having an equal impact across NZ.

#### Objective 1.1: quantify change in levels of participation

Change in the percentage of residents who have participated in local alcohol decision-making (e.g., attending a public meeting, making a submission on a draft policy, objecting to a license application) will be measured. Reasons for participating or not participating will be examined. The opinions of participants versus non-participants will be compared to determine whose voices are being heard.

Cross-sectional and longitudinal national surveys will be conducted. The first was undertaken in 2014 and the second will be completed in 2017 (detailed below). Questions were derived from a survey conducted in seven TAs [24, 25], that asked residents about their involvement in local alcohol decision-making. The 2014 survey asked if any participation since 2013 was related to LAP development. This will allow us to estimate levels of participation before the SSAA was introduced. The 2017 survey will ask about participation with reference to the previous 3 years.

χ^2^ tests will be used to examine change in participation levels. We will summarise residents’ motives for participating and regression analyses will be used to identify correlates of participation and examine the difference in pre-post change between Māori and non-Māori.

#### Objective 1.2: quantify change in the number of objections per license application

The number of objections per licence application before and after the SSAA came into effect will be determined. Data from July 2003 to December 2016 will be obtained from TA District Licensing Committees (DLCs) and the Alcohol Regulatory and Licensing Authority (ARLA). It is anticipated that negative binomial regression (for skewed count distributions) will be used to analyse the data.

It is possible that residents will feel disempowered by local decision-making processes regardless of the new law and not object to a licence application despite being against it. Alternatively, they may be happy with the actions of decision-makers. We will use the national survey data to examine these possibilities.

#### Objective 1.3: describe the process by which LAPs have been developed

*Interviews with iwi.* Māori may participate in decision-making individually and/or collectively as iwi (i.e., ‘people’ or ‘nation’ but often translated as ‘tribe’) [26]. TAs are required by law to provide for Māori participation in local decision-making processes [27]. Eight iwi will be asked about the role they see themselves playing in responding to alcohol issues in their communities, the level of involvement and influence they would expect to have over local decisions, their experience with local government consultation on alcohol policy, whether they feel this has improved subsequent to the SSAA being introduced, and the level of influence they believe they have had over decisions. Data will complement the national surveys, providing another perspective on Māori participation in local alcohol decision-making.

Iwi will be purposively selected and invited to take part in the study. The two iwi that made submissions on the Alcohol Reform Bill will be invited to participate and other iwi will be selected to ensure broad geographical representation.

*Interviews with community groups.* Community groups from eight TAs that have taken action on local alcohol issues under the old and new legislation will be asked about their experience of decision-making processes, the influence they think they have had on policy and/or licensing outcomes, and whether they believe this has improved under the SSAA.

Community groups will be identified through the development of alcohol policy histories. As in our previous research [28], these histories will be established by searching regional newspapers for articles in the past 10 years pertaining to alcohol. Groups invited to participate will be selected to ensure geographical and socio-demographic variety. Group representatives will be contacted by mail and a follow-up telephone call and invited to participate in a 1 h face-to-face or telephone interview.

*Interviews with TA staff.* We will determine how TAs have engaged the public, iwi and interest groups (e.g., neighbourhood collectives, industry groups) in decisions on LAP adoption and content. We will establish how consultation methods changed. Reasons why a LAP has or has not been adopted, factors impeding or facilitating adoption, and elements that have influenced policy content will be investigated.

#### Objective 1.4: determine the number and characteristics of TAs developing LAPs by 2017

The number of TAs in NZ that have adopted or are developing LAPs by 2017 will be determined. Census 2013 data will be used to identify socio-demographic factors (at the TA level) associated with LAP adoption and restrictiveness. This will reveal whether particular sectors of society (e.g., people living in relatively deprived communities) are missing the opportunity to have input or are being exposed to environments more conducive to hazardous drinking [29].

LAP adoption will be ascertained from ARLA. Annual telephone interviews with local government staff responsible for alcohol policy will be conducted to corroborate this information or otherwise determine the status of a LAP.

Logistic regression will be used to investigate correlates of LAP adoption, including population size, NZ Deprivation Index Score (population-weighted) [30], and the percentage of the population that is Māori. We will use an urban/rural index and a measure of TA resources (using annual reports) and examine their associations with LAP adoption.

Assuming the rule of thumb of ten cases per independent variable[31] we will need ≥10 TAs to have adopted a LAP by 2017 (but ≤57 as this would mean there were <10 ‘not adopted’ cases) to produce stable coefficient estimates. Consultation with local government staff indicates that considerably more than 10 LAPs will have been adopted by then. If more than 57 TAs have adopted a LAP the outcome measure will be how restrictive the LAP is compared to the TA’s previous alcohol controls, i.e., more restrictive versus no change/less restrictive.

### Part 2: change in alcohol availability

Part 2 addresses the second aim: evaluating the effect of the SSAA on alcohol availability. This will be assessed in statutory and physical terms.

#### Objective 2.1: quantify how alcohol availability has changed in statutory terms

Local government staff will be asked in telephone interviews how alcohol policy has changed since the new law was introduced. Interview data will be supplemented with data obtained via analysis of policy documents. This will allow us to determine whether policies in place in each community (via a LAP or the new default hours prescribed in the legislation) are more restrictive, more liberal, or no different to policies in place prior to the law change. We will determine the number of TAs where maximum permitted closing times for on-license outlets, off-license outlets, clubs and restaurants, and the use of one-way door restrictions have reduced, stayed the same or increased.

#### Objective 2.2: quantify how alcohol availability has changed in physical terms

We will examine change in the number (per 1000 residents) and density (per square kilometre) of alcohol outlets after the SSAA takes effect. This will be measured by updating a geographic information system (GIS) developed by members of the research team [32]. The GIS maps the location of every liquor license, by license type (i.e., pub/bar, bottle-store/supermarket, club, restaurant) in NZ since 1995. Previous analyses showed that access to alcohol outlets was greater in more deprived areas [32]. We will examine whether and how this relationship has changed after the new law took effect. Change in the total number of weekend late-night trading hours will also be examined in each TA.

Data from the Ministry of Justice will be used to update the GIS to 31 December 2016 and calculate weekend trading hours for each active outlet. Change in the number and density of outlets (by license type) and in the number of weekend late-night (i.e., post 10 pm) trading hours will be modelled using negative binomial or Poisson regression. These analyses will estimate the relative change in the number and density of alcohol outlets, and total number of weekend late-night trading hours.

Weekend late-night trading hours will be quantified by summing the number of hours past 10 pm that each outlet is open on Friday night/Saturday morning and Saturday night/Sunday morning. This analysis will be conducted separately for on-license and off-license premises.

### Part 3: estimate change in alcohol consumption and related harm

Part 3 addresses the third aim: to evaluate the effect of the new laws (SSAA and BAC limit change) on alcohol consumption and alcohol-related harm.

#### Objective 3.1: quantify change in the prevalence of hazardous drinking, personal and secondhand harms, and community amenity

We will investigate how the prevalence of hazardous drinking, problems experienced from one’s own drinking, secondhand effects [33] (i.e., harms due to other’s drinking) and community amenity effects [34] change. The 2017 national survey, as in 2014, will measure hazardous drinking using the AUDIT [35]. Additional questions will again be asked about physical aggression [36] and driving under the influence of alcohol [37]. The proportion of residents who have experienced various secondhand and community amenity effects will be estimated using scales we have employed previously [33, 34].

The laws could affect where and how much people drink in different locations. This may result in a change in rates of harm but no change in the prevalence of hazardous drinking. We have therefore included questions from previous population surveys [38] on the amount of alcohol consumed in different contexts. To account for changes in drinking patterns, we will again ask questions in 2017 on the frequency of very heavy drinking occasions (i.e., ≥200 g ethanol) [39].

*χ* ^2^ tests will be used to examine change (in Māori, non-Māori, and combined samples) in the prevalence of hazardous drinking, adverse effects of respondents’ own drinking, secondhand effects and community amenity effects. Regression analysis will be used to examine the difference in pre-post change between Māori and non-Māori.

#### Objective 3.2: estimate change in rates of assault

Change in the incidence of late-night assault (10 pm-6 am) apprehensions by police and weekend hospitalisations resulting from assault will be estimated. All weekend hospitalisations for assault will be included because alcohol involvement in assault is not consistently established by hospital staff [40], and the time an assault occurred is not routinely recorded in hospital discharge data [41].

We will use de-identified data on police apprehension for assault (as defined in the New Zealand Crimes Act 1961) [42] from July 2003 to December 2016. Data on weekend assault hospitalisations will be obtained from the National Minimum Dataset of hospital discharges [43]. We will obtain information from July 2003 to December 2016 on the number of individuals with an ICD-10 external cause of injury code referring to assault who were hospitalised for at least one night.

Negative binomial or Poisson regression will be used to estimate the relative change in the incidence of late-night assault apprehensions and weekend assault hospitalisations.

#### Objective 3.3: quantify change in the rate of alcohol-involved traffic crashes

Traffic Crash Reports submitted by Police include breath or blood alcohol test results and in their absence, the attending officer’s assessment of whether alcohol was involved. The subjective assessments have been shown to be reasonably accurate [44]. Poisson regression will be used to estimate change in the incidence of alcohol-involved traffic crashes.

### National surveys

The first national survey was conducted in the second half of 2014 and the second will be conducted in the second half of 2017. The surveys are based on our previous research into public sentiment and local government alcohol policy in NZ [24]. This postal survey involved seven TAs and had a 59 % response fraction [24], informing our sample size calculation. The survey protocol follows procedures found in systematic reviews to maximise participation [45]. The principle of Mana Whakamārama [46] (equal explanatory power) will be applied to produce knowledge of sufficient breadth and depth for Māori. An invitation letter, information sheet, questionnaire and postage-paid return envelope was mailed to 4000 residents randomly selected from the electoral roll (2000 Māori Roll/2000 General Roll) throughout NZ in September 2014. A reminder letter was sent 3 weeks after the initial mail-out to those who had not responded. A second reminder letter and replacement questionnaire was posted to those yet to respond after a further 4 weeks and a final reminder postcard was sent after a further 3 weeks. The act of returning the questionnaire fully or partially completed was taken as consent to participate. This procedure will be followed in 2017.

Being registered on the electoral roll is compulsory in NZ. Latest estimates suggest 92 % of eligible voters are enrolled [47]. Citizens who are the descendent of a NZ Māori may choose to be on either the Māori or General electoral roll. Approximately 7 % of electors on the General roll (as at August 2014) indicate they are of Māori descent but it is possible that not all of these individuals identify as being of Māori ethnicity or are strongly connected to Te Ao Māori (“the Māori World”) in terms of familiarity with Māori language, protocols and customs.

In 2017 the questionnaire will be mailed to those who responded in 2014 as well as a new sample of 4000 residents. The new sample will allow us to estimate the effect of attrition and research participation effects [48] in the longitudinal sample while having the longitudinal data will allow us to examine more precisely who the law is affecting.

A 50 % response rate in 2014 and in the new sample in 2017 (i.e., the independent samples) would provide >80 % statistical power with alpha of 5 % to detect a true difference of 6.5 % between survey waves in the outcomes of interest in both the Māori and General roll samples. For the longitudinal sample, assuming attrition of 20 % in 2017 and item response correlation of 0.5 between waves (a conservative estimate for consumption items) [49], the response rate would provide 80 % power for the Māori and General roll samples to detect a true difference of 5 % between survey waves. These are conservative estimates based on a prevalence estimate of 50 %. For most outcomes statistical power will be greater.

### Interviews with iwi and community groups

The experience of iwi and community groups with local government consultation on alcohol issues and participation in decision-making will be investigated. Data will be collected using face-to-face interviews. These will be semi-structured, allowing iwi and community group representatives to raise issues about consultation and decision-making processes that are of greatest importance to them while also allowing us to cover particular issues should these not be raised naturally during the interviews (e.g., influence they feel they have had, if the SSAA has facilitated input, and barriers to participation). The written consent of participants will be obtained prior to commencing the interview. Interviews will be recorded with participants’ permission and transcribed for analysis.

### Interviews with territorial authority staff

The CEO of each TA will receive a letter informing them of the study and requesting the names and contact details of staff involved in the development of alcohol policy. These staff will be sent an information sheet about the study and invited to participate in a telephone survey. Consent will be obtained via email and/or a follow-up phone call once any questions the staff member has have been answered. An interview time will then be arranged if they are happy to participate. Phone interviews will be conducted annually until 2017 using a semi-structured questionnaire [28]. The staff member will be reminded before commencing the interview of details outlined in the information sheet: that they can refuse to answer any question, withdraw from the interview and study at any time, and that every effort will be made to preserve their anonymity in anything that we publish or present. They will then be asked again if they are happy to proceed. Local government documents relevant to decision-making on LAPs will be requested at the conclusion of interviews. Recorded interviews and relevant policy documents will be analysed thematically [50].

### Analysis

Analyses of the national survey data will be conducted on the Māori roll sample, General roll sample and the combined sample. The data will be post-weighted when analysing the combined sample to adjust for the oversampling of Māori. We will conduct separate Māori analyses on archival data if feasible, but it is likely that limited statistical power (Māori comprise only 15 % of the NZ population) and/or problems inherent to the data (e.g., incomplete or missing data, subjective assessment of ethnicity) will restrict our ability to do this.

The only other statistical analyses likely to be constrained by statistical power are the logistic regression analyses at the TA level (where *n* = 67) to identify correlates of LAP adoption. All other statistical analyses (i.e., national survey and archival data) will have sufficient power to detect modest changes in the outcomes of interest [8, 51].

Negative binomial and Poisson regression analyses of the archival data will model the effect of the law change using a pre-post dummy variable. These have been chosen over interrupted time-series analysis as it is anticipated that the rates and counts of interest will be skewed towards zero. A continuous time variable and categorical quarter variable will be used in the regression analyses to adjust for secular and seasonal trends. Estimated population figures from Census data will be used to produce incidence rates and to adjust for change in population size over time. Autocorrelation will be tested for and models will be adjusted accordingly.

### Pre-law period

**Fig. 2:**
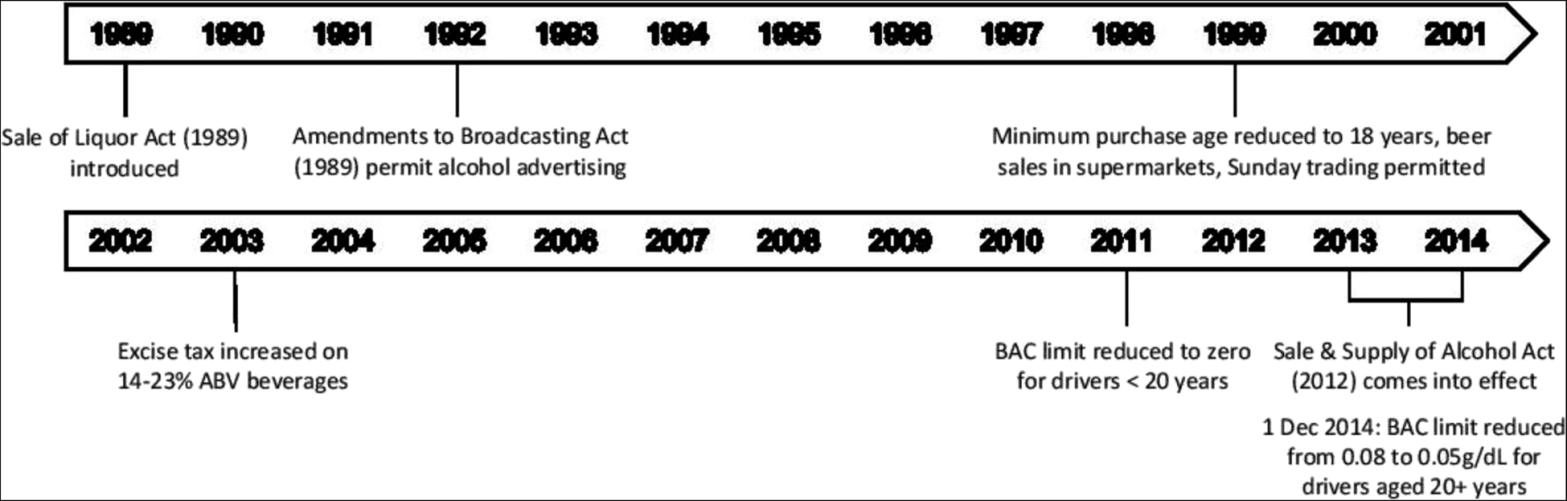
Timeline of alcohol legislation changes

The pre-law period will be 1 July 2003 to 31 May 2013, beginning shortly after a law change on 7 May 2003 that increased the excise tax on alcoholic beverages with 14–23 % alcohol-by-volume to be the same as that of beverages with >23 % alcohol-by-volume (Fig. 2). Based on previous research we have assumed the effect of this change to have been immediate [52], and small in the context of our 10 year pre-law data series. The pre-law period also includes an amendment to the Land Transport Act which saw the legal BAC limit for drivers aged <20 years reduced from 0.03 g/dL to zero. In the context of our analyses—of the whole population—we anticipate the impact of this to have been minor but we will nevertheless examine the impact of this law change separately for younger drivers and take the findings into account in our overall analysis.

### Post-law period

The post-law period for the analysis of objections to license applications will be 1 July 2013 to 31 December 2016. For the analyses of alcohol availability it will be 18 December 2013 to 31 December 2016. For analyses of rates of alcohol-related harm, the law changes (SSAA introduction and BAC limit change) will be treated as a single package meaning the post-law period will be 1 December 2014 to 31 December 2016. These time points allow for implementation of the SSAA in the months between the pre- and post-law periods while providing a post-law time period that allows the effect of the laws to be assessed.

## Discussion

We have designed a study that will evaluate how well NZ’s new alcohol laws meet their explicit public health objectives, to the extent possible given limitations of data and the timing of the grant funding relative to the law change. The study is, however, subject to limitations. Changes in public participation in local government decision-making on alcohol, drinking behaviours and alcohol-related harms will be measured using postal surveys. We estimate a response fraction of 50 %, resulting in a sample that is not representative of the underlying population. We will assess non-response bias in each survey by comparing participants versus non-participants and early versus late respondents on a range of variables [53].

The baseline survey took place from September to December 2014. During this time only two LAPs were in force, both in rural communities. New drink-driving limits came into effect on 1 December 2014 by which time 9 out of 10 respondents had returned their questionnaires. Accordingly, the survey largely reflects drinking behaviour and alcohol-related harm prior to the law changes, however, there remains a possibility of bias in estimates of change from pre- to post-intervention, toward the null. Similarly, media coverage leading up to the drink-driving law change may have led some people to believe the new limit was in place prior to 1 December 2014. Estimates of change would be biased toward the null if the population reduced its drink-driving before the law came into effect.

Another potential limitation is error in police data. These are subject to changes in service delivery, e.g., because of changes in the number of police patrolling the streets, which may change the detection of assault without affecting the incidence [54].

Inferences about the specific effects of a policy applied to an entire population are risky because observed changes may be due to extraneous factors. For example, alcohol consumption may decrease because of an economic downturn [55]. All three parts of this study are therefore vital to gain information on the implementation of the SSAA, changes in alcohol availability, and changes in the incidence of various harms. This will provide insight that enables testable hypotheses to be generated. For example, if most TAs adopt LAPs that are more restrictive than their previous policies, yet we find no change in alcohol availability, this would suggest decision-makers are not adhering to LAPs or utilising the powers available to them under the Act. We may, nonetheless, find a decrease in rates of hazardous drinking and alcohol-related harm, perhaps due to a reduction in alcohol consumption because of economic factors. We will attempt to adjust for such factors in our analyses by controlling for the unemployment rate and/or relative differences between growth in salaries and the consumer price index.

It should be noted that the limitation arising from having no suitable control population against which to compare the effects of the new laws applies only to the third aim and will be offset with the examination of change as a function of LAP adoption and use of statistical control for known confounders.

Current research evidence does not suggest that the law reform package will have a significant effect on rates of community participation in licensing decisions, the availability of alcohol in NZ, or the prevalence of hazardous drinking and alcohol-related harm [12, 56]. Nonetheless, we strive to be impartial and seek to prevent confirmation bias [57] by pre-specifying study aims, methods, and analysis plans, using robust protocols that have been used previously and peer-reviewed. Qualitative interviews will be semi-structured to allow participants to raise issues of greatest importance to them. When analysing data we will search for and present evidence that is contrary to expectations, and we will test competing explanations for what we observe. The research team is multi-disciplinary and multi-national and we will use it and external advisors to help identify flaws in our methods and alternative explanations for the results.

### Relevance to health and policy

The research will generate knowledge on the uptake and development of LAPs and whether public input into local decision-making on alcohol changes. Currently there is no empirical evidence on whether increasing community input into local licensing decisions and broadening licence refusal criteria is effective in reducing alcohol availability, a major determinant of hazardous drinking [4]. A number of factors can obstruct public participation, including a lack of knowledge, low self-efficacy, a lack of resources; and cultural or political barriers [58]. Political structures may also create or maintain power imbalances between groups that do not result in responsive governance [28, 58, 59]. If the law is to be effective we should see a reduction in alcohol availability within the 4 years of this project. This study will inform policy-makers about the potential of this novel strategy to be an effective component of healthy alcohol policy in NZ and other countries. In addition, it will increase understanding of how local government considers, develops, adopts and implements alcohol policy, which has received little attention to date [60].

The research also investigates whether relatively deprived communities are exposed to greater availability and promotion of alcohol under the new legislation. This research will contribute to addressing the health needs of Māori by identifying the level of Māori engagement in the development of Local Alcohol Policies, and their ability to influence local alcohol availability, establishing whether Māori are getting an equal say in where, when and how alcohol is sold in their communities. It will also assess the impact of the new legislation on alcohol consumption and related harm among Māori.

### Future research

The study will provide a substantial set of baseline data for evaluating future changes in alcohol legislation. It will provide a foundation for evaluating the effectiveness of LAPs and we will compare pre-post change in harm among those exposed versus unexposed to an LAP. Our ability to do this within the current study will depend on when LAPs become operational. This analysis will allow partial control for effects of the BAC law change and extraneous factors (e.g., economic conditions).

It is possible that changes will take longer to manifest than the post-law periods of this evaluation. Should we see no changes within the next few years, the study protocol allows for the evaluation to be extended, including repetition of the national survey and collation of further administrative data. If change is evident early, evaluation of the longer term impacts would still be desirable to determine whether changes are sustained, particularly given LAPs have to be reviewed every 6 years and can be changed or revoked at any time, subject to community consultation [13].

## Abbreviations

ARLA: Alcohol Regulatory and Licensing Authority
AUDIT: Alcohol Use Disorders Identification Test
BAC: Blood alcohol concentration
DALYs: Disability adjusted life years
DLC: District licensing committee
ENZAL: Evaluation of New Zealand’s Alcohol Laws
GIS: Geographic information system
LAP: Local alcohol policy
NZ: New Zealand
SSAA: Sale and Supply of Alcohol Act
TA: Territorial authority
